# Oxidative Stress Response in *Pseudomonas aeruginosa*

**DOI:** 10.3390/pathogens10091187

**Published:** 2021-09-14

**Authors:** Waleska Stephanie da Cruz Nizer, Vasily Inkovskiy, Zoya Versey, Nikola Strempel, Edana Cassol, Joerg Overhage

**Affiliations:** 1 Department of Health Sciences, Carleton University, Ottawa, ON K1S 5B6, Canada; waleskadacruznizer@cmail.carleton.ca (W.S.d.C.N.); VasilyInkovskiy@cmail.carleton.ca (V.I.); zoyaversey@cmail.carleton.ca (Z.V.); edanacassol@cunet.carleton.ca (E.C.); 2 Institute of Functional Interfaces, Karlsruhe Institute of Technology, 76344 Karlsruhe, Germany; nikola.strempel@alumni.kit.edu

**Keywords:** *Pseudomonas aeruginosa*, oxidative stress, oxidative stress response, reactive oxygen species, reactive chlorine species, antimicrobial resistance, bacterial stress response

## Abstract

*Pseudomonas aeruginosa* is a Gram-negative environmental and human opportunistic pathogen highly adapted to many different environmental conditions. It can cause a wide range of serious infections, including wounds, lungs, the urinary tract, and systemic infections. The high versatility and pathogenicity of this bacterium is attributed to its genomic complexity, the expression of several virulence factors, and its intrinsic resistance to various antimicrobials. However, to thrive and establish infection, *P. aeruginosa* must overcome several barriers. One of these barriers is the presence of oxidizing agents (e.g., hydrogen peroxide, superoxide, and hypochlorous acid) produced by the host immune system or that are commonly used as disinfectants in a variety of different environments including hospitals. These agents damage several cellular molecules and can cause cell death. Therefore, bacteria adapt to these harsh conditions by altering gene expression and eliciting several stress responses to survive under oxidative stress. Here, we used PubMed to evaluate the current knowledge on the oxidative stress responses adopted by *P. aeruginosa*. We will describe the genes that are often differently expressed under oxidative stress conditions, the pathways and proteins employed to sense and respond to oxidative stress, and how these changes in gene expression influence pathogenicity and the virulence of *P. aeruginosa*. Understanding these responses and changes in gene expression is critical to controlling bacterial pathogenicity and developing new therapeutic agents.

## 1. *Pseudomonas aeruginosa* Pathogenicity and Virulence

*Pseudomonas aeruginosa* is a ubiquitous environmental Gram-negative bacterium. It causes several hospital-acquired infections, such as severe systemic infections, endocarditis, ventilator-associated pneumonia, and urinary tract infections [1,2]. As an opportunistic pathogen, *P. aeruginosa* successfully causes infections in immunocompromised individuals, patients on mechanical ventilation, elderly people, or patients with comorbidities. [3]. Furthermore, *P. aeruginosa* is considered to be the leading cause of chronic lung infections in cystic fibrosis (CF) patients, often resulting in lung failure [4,5,6]. In particular, multidrug-resistant (MDR) *P. aeruginosa*, which causes more than 50,000 healthcare-associated infections in the United States annually, is considered an emerging threat to human health [7].

The pathogenicity and success of *P. aeruginosa* as a human and environmental pathogen are attributed to several factors, including (i) its ability to develop adaptive and acquired resistance mechanisms, as well as its intrinsic resistance to several classes of antibiotics and disinfectants [8]; (ii) the expression of an arsenal of virulence factors [9]; and (iii) its ability to thrive in different environmental conditions, such as water with very low nutrient content [10].

*P. aeruginosa* is intrinsically resistant to many commonly used antibiotics due to low outer membrane permeability, which impairs drug influx. [11]. Active efflux pumps also promote the rapid transport of toxic compounds through the membrane [12,13]. Furthermore, the expression of antibiotic-cleaving enzymes also confers resistance to several classes of antibiotics. For instance, *P. aeruginosa* is inherently resistant to β-lactam antibiotics, such as penicillins and narrow-spectrum cephalosporins, due to the expression of the β-lactamase AmpC [12].

In addition to the intrinsic resistance to several classes of antibiotics, *P. aeruginosa*, like many other bacteria, can also acquire antibiotic resistance via horizontal gene transfer of plasmid-encoded resistance determinants and genetic mutations [11,12,13]. The development of such mutational resistances is favored by the direct exposure of bacteria to DNA-damaging substances [13], as is the case with different disinfection by-products or antibiotics [14,15], which may lead to the permanent overexpression of resistance-associated genes, such as multidrug efflux pumps [11]. In contrast to intrinsic and acquired resistances, which are usually stable, inheritable, and in general independent of the environment, adaptive resistance mechanisms are specifically induced under certain environmental conditions. They are characterized by changes in gene expression patterns leading to a temporary increase in antimicrobial resistance. An important example of adaptive resistance is the formation of biofilms [11].

In addition to antimicrobial resistance, the pathogenicity of *P. aeruginosa* is also attributed to the expression of multiple virulence factors. These factors comprise: the expression of (i) surface-bound flagella and type IV pili, which are responsible for bacterial motility and adhesion to a surface [16,17,18]; (ii) the type III secretion system (T3SS), a needle-like complex that allows the direct injection of the toxic effector proteins ExoY, ExoS, ExoT, and ExoU into host cells [19]; (iii) secreted exocompounds, such as proteases, exotoxins, pigments, and rhamnolipids [20,21,22]; (iv) alginate, which is the main component of the capsule that characterizes the mucoid phenotype and represents a common phenotype in chronic CF lung infections [23,24]; and (v) lipopolysaccharide (LPS), which represents a physical barrier against antimicrobials [25]. Most of these virulence factors contribute to the pathogenicity of *P. aeruginosa* by enabling the pathogen to invade and compromise the host immune responses. For instance, pyocyanin has been reported to induce tissue damage by penetrating biological membranes, inactivating certain enzymes, and inducing apoptotic cell death of neutrophils and epithelial cells via the formation of reactive hydroxyl radicals [26].

The formation of biofilms is not only considered an important virulence factor, but it is also involved in antimicrobial resistance. Biofilms are microbial communities attached to surfaces that serve as protection against harsh environmental conditions. The formation of these sessile structures is induced by diverse factors, such as pH, oxygen availability, and cellular metabolites [27,28]. In this context, studies have shown that *P. aeruginosa* biofilm formation is stimulated by the presence of sublethal concentrations of antimicrobials, such as the aminoglycosides tobramycin and gentamicin [29,30], β-lactam imipenem [31], the fluoroquinolone ciprofloxacin [30], the disinfectant sodium hypochlorite [32], and the detergent sodium dodecyl sulfate (SDS) [33] (reviewed in [34]). This finding may be of particular clinical interest since sublethal concentrations of antimicrobials have been reported to be used at the beginning or the end of antibiotic therapy [34,35]. Antibiotic resistance mechanisms of *P. aeruginosa* have previously been described in excellent reviews [11,15,36].

Despite its pathogenicity and adaptability to diverse environmental conditions, *P. aeruginosa* is continuously confronted by oxidative stress in its natural environments, whether produced endogenously via respiration or exogenously by the host immune system or disinfectants. These agents react with several cellular molecules and can cause irreversible damage to the bacteria, leading to cell death. Therefore, bacteria have developed an arsenal of adaptive mechanisms to respond to and manage oxidative stress damage. In this article, we will start by describing reactive oxygen and chlorine species (ROS and RCS, respectively), their mechanisms of action, and where bacteria encounter them. We will then summarize the currently available knowledge on the strategies adopted by *P. aeruginosa* to counteract the toxic effects of oxidizing agents. We will describe cellular responses, such as the induction of a mucoid phenotype and production of detoxifying enzymes, as well as the activation of transcriptional regulators and induction of antibiotic resistance by oxidative stress.

## 2. Oxidative Stress

Oxidizing agents are low molecular-weight molecules that can easily penetrate the membrane or cell wall of bacterial cells and cause intracellular damage. Due to their antimicrobial effect, many oxidizing agents have been used to eradicate pathogens [37,38]. Among the most common examples of oxidizing agents, ROS and RCS, such as hydrogen peroxide (H_2_O_2_) and hypochlorous acid (HOCl), are the most widely used disinfectants. Furthermore, they are also produced by the host immune system as a defense against invading pathogens and can also be endogenously produced by bacteria [38]. Although the reactivity of these toxic oxygen species can vary, they share many common modes of action, including the oxidation of (i) proteins, which leads to the disruption of protein and DNA synthesis and enzyme activity; (ii) lipids, leading to membrane destabilization; and (iii) nucleic acids, causing DNA breaks [37,39].

### 2.1. Reactive Oxygen Species

**Figure 1:**
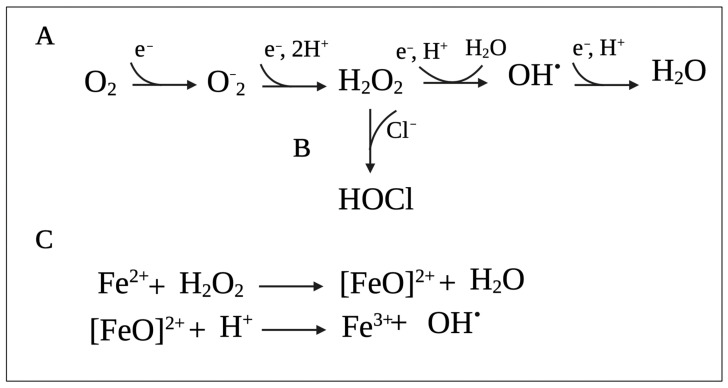
Series of reactions leading to the production of (**A**) reactive oxygen species (ROS) from molecular oxygen (O_2_) and (**B**) hypochlorous acid (HOCl). (**C**) Fenton reaction between ferrous iron (Fe^2+^) and hydrogen peroxide (H_2_O_2_) leading to the production of hydroxyl radical (HO^•^). Superoxide: O_2_^−^, hydrogen peroxide: H_2_O_2_, hydroxyl radicals: HO^•^, H_2_O: water, e^−^: electron, H^+^: hydrogen ion. This figure was created with BioRender.com (accessed on 10 September 2021).

Reactive oxygen species (ROS) (i.e., superoxide (O_2_^−^), H_2_O_2_, and hydroxyl radicals (HO^•^)) represent the most common groups of oxidizing agents, which are formed when molecular oxygen (O_2_) acquires single electrons (Figure 1A) [40,41]. These toxic oxygen species can be produced by the bacteria or by the host immune system or can be found in the environment as disinfectants and antimicrobials.

In bacterial cells, flavoprotein enzymes are believed to be the primary source of endogenous O_2_^−^ and H_2_O_2_ [42,43]. It was shown that ROS is primarily generated by the autoxidation of reduced flavoproteins not involved in the respiratory chain since bacterial mutants lacking respiratory components also produced ROS at comparable rates [40,44]. Examples of these enzymes include NADH dehydrogenase II, used in the aerobic respiratory chain as a primary site of electron transfer to oxygen, and fumarate reductase, which is a terminal oxidase active under anaerobic conditions [45]. Fumarate reductase reacts rapidly with oxygen and is believed to be the major source of oxidative stress when facultative anaerobes enter aerobic environments [40,45]. Importantly, since autoxidation depends on the collision frequency between enzymes and oxygen, higher oxygen concentrations lead to increased ROS production [44].

Studies also suggest that in addition to the traditional mechanisms of action, antibiotic lethality is also mediated (at least in parts) by ROS produced due to alterations in bacterial metabolism, respiration, and iron homeostasis [46]. For example, one study in *P. aeruginosa* showed that exposure to antibiotics accelerated cell death by promoting the Fenton reaction (Figure 1C), in which HO^•^ is generated by the reaction between ferrous iron (Fe^2+^) and H_2_O_2_ [47]. Additionally, the generation of ROS due to the exposure of *E. coli* to sublethal concentrations of antibiotics increases their mutation rates due to oxidative damage of DNA and leads to the emergence of MDR [48]. Moreover, Foti et al. (2012) proposed that antibiotic lethality results from a failure to repair closely spaced oxidized nucleotides 8-oxo-deoxyguanosine (8-oxo-dG), leading to the formation of double-stranded breaks [49]. Despite this evidence, exact mechanisms of ROS-dependent antibiotic killing are currently unknown [46].

In addition to the endogenous production of ROS, bacteria are also exposed to exogenous oxygen species. For example, environmental H_2_O_2_ can be produced during the chemical oxidation of reduced metals and sulfur at oxic-anoxic surfaces, the reduction of molecular oxygen by flavins and chromophores, the excretion of H_2_O_2_ by lactic acid bacteria, as well as during H_2_O_2_ production by NADPH oxidases in mammalian and plant phagocytes [38]. In this context, the stimulation of phagocytes by phagocyte particles, such as bacteria, induces an increase in oxygen use, leading to the release of toxic oxygen species. For instance, the concentration of H_2_O_2_ during bacterial phagocytosis was increased by more than 50 times compared to basal conditions, in which the concentration of H_2_O_2_ was lower than 0.1 nmol/mL per min [50]. Furthermore, it was shown that 10^7^ leukocytes produce approximately 1.03 nmol of O^−^_2_ in 15 min [51]. As a disinfectant, a concentration ranging between 5–35% (*w*/*w*) is recommended [52].

### 2.2. Reactive Chlorine Species

Reactive chlorine species (RCS) are comprised of a group of highly reactive compounds that are capable of oxidizing and chlorinating other molecules and include HOCl and chloramines (R_2_NCl) [53]. Among these, HOCl is the most potent oxidant that easily penetrates bacterial membranes and reacts with most cellular molecules, including sulfur-containing compounds (cysteine, methionine, glutathione), primary and secondary amines, nucleotides, and lipids. A summary of oxidative HOCl reactions and microbial responses to HOCl-induced stress were previously summarized in other reviews [32,53]. The main ingredient of chlorine-based bleach is sodium hypochlorite (NaOCl), which is converted into HOCl in aqueous solution [54].

RCS represent a group of oxidizing agents frequently encountered by bacteria in their native habitats [53]. One of the major sources of RCS in the environment is NaOCl, which is the main ingredient of the most commonly used chlorine-based disinfectant, bleach [32]. Bleach is frequently employed for disinfection purposes in industrial, hospital, and household settings, as well as for water disinfection and wastewater treatment [32,55]. NaOCl is only one of the forms of free chlorine in the aqueous solution, while the other forms include chlorine gas (Cl_2_), HOCl, and hypochlorite ion (^−^OCl). The relative concentrations of different forms of chlorine depend on the pH of the solution. At higher pH values (i.e., 8.510), most of the chlorine in solution is from ^−^OCl, while at pHs between 4 and 6, the prevalent species is HOCl, and at acidic pH (lower than 4), the chlorine concentration found in solution is mostly due to the presence of Cl_2_ [18,48]. Out of these chlorine species, HOCl has the greatest microbicidal action [54].

### 2.3. Oxidative Stress Generated by the Host Immune System

ROS play an essential role in the host’s innate immune response to microbial pathogens. The main sources of ROS are professional phagocytes, such as neutrophils and macrophages, which are recruited to the site of infection to support early pathogen elimination. They recognize and bind bacterial pathogen-associated molecular patterns (PAMPs) through pattern recognition receptors (PRRs) [56], which activate various microbicidal functions, including the release of inflammatory mediators, the formation of neutrophil extracellular trap (NET), and degranulation [57,58]. It also results in increased phagocytosis and ROS production, which supports bacterial uptake and intracellular killing [53,54]. Microbes and microbial products encounter high concentrations of ROS when they are phagocytosed into intracellular compartments called phagosomes [59,60].

Although PMNs are essential in eliminating *P. aeruginosa*, in CF patients, they do not clear the infection since this pathogen is known to adapt to their killing mechanisms, such as by avoiding phagocytosis and forming biofilms [61]. Furthermore, the formation of biofilms and a marked inflammatory phase, which includes oxidative damage, in chronic wound infections are associated with poor outcomes [62,63]. In this context, the production of antimicrobial peptides produced by the skin’s innate response plays essential roles in the immune response of chronic wounds. However, the presence of *P. aeruginosa* biofilms in these infections reduces neutrophil response [62].

The two major systems that generate ROS in immune cells are the NOX2 NADPH oxidase multicomplex and mitochondrial ROS. The NOX family of NADPH oxidases is comprised of membrane-bound isoforms NOX1-5 and DUOX1-2 [64]. The NOX2 NADPH oxidase multicomplex plays a central role in this process by mediating the rapid release of ROS during an immune response, described as respiratory burst. These components assemble in the phagosome membrane to form a multicomplex [65] in response to phagocytosis. Simultaneously, azurophilic granules fuse with the phagosome to release myeloperoxidase (MPO) in the phagosome space [65]. NOX2 catalyzes the reduction of O_2_ into O_2_^−^, which accumulates in the phagosome [66]. The current understanding is that O_2_^−^ serves as an intermediate molecule and is immediately converted to H_2_O_2_ spontaneously by superoxide dismutases (SOD) [65,66]. In addition to NOX2, the mitochondrial electron transport chain (ETC) is a key site for ROS generation. Engagement of cell surface TLRs, TLR-1, -2, and -4 by bacterial ligands lipopeptide, lipoteichoic acid, and LPS, respectively, have been shown to activate the migration of mitochondria to the phagosome membrane in macrophages [67]. ROS is primarily produced when electrons leak from ETC complexes I and III and reduce O_2_ and generate O_2_^−^ [68]. Mitochondrial-derived vesicles containing superoxide dismutase-2 (SOD2) are delivered to the phagosome, where mitochondrial O_2_^−^ dismutases to H_2_O_2_ [68,69]. Together, the NOX2 and the mitochondrial ETC amplify H_2_O_2_ production within the phagosome.

In addition to mediating antimicrobial effects, H_2_O_2_ also serves as a substrate for RCS generation. H_2_O_2_ can be oxidized by ferrous iron to generate HO^•^ [66]. However, the phagosome holds high Cl^−^ concentrations [70]. As such, it is accepted that most of the H_2_O_2_ is catalyzed by MPO to generate HOCl (Figure 1B) predominantly, and to a lesser extent, HOSCN [71,72,73,74]. These products directly kill the ingested bacteria. In comparison to H_2_O_2_, HOCl requires lower concentrations to cause lethal effects in bacteria [53]. For instance, it was shown that 0.2 mmol of HOCl generated by approximately 10^6^ stimulated neutrophils can destroy as many as one million *E. coli* cells in an extremely short time [75]. Interestingly, Winterbourn et al. [65] have proposed that before HOCl can reach the bacteria, it targets phagosomal proteins to form chloramines. It may seem counterintuitive, but small chloramines, e.g., monochloramine (NH_2_Cl), have the potential to diffuse into bacterial cells and exert cytotoxic effects [67]. To counteract this issue and improve the effectiveness of HOCl, MPO can selectively bind to cells of some bacterial species to drive HOCl to its intended target [76].

### 2.4. Mechanisms of Oxidative Cell Damage

Oxidizing agents share many cellular targets, such as thiol groups of proteins, nucleotide bases, and lipids, which can affect ATP, DNA, and protein synthesis, enzyme activity, and membrane integrity [37]. An important aspect of the microbicidal activity of HOCl and ROS is their ability to easily penetrate bacterial membranes due to their neutrality. Inside bacterial cells, they can react with many targets and disrupt multiple cellular processes [40,54]. They can also work in concert to drive bacterial killing [77]. Specifically, studies in *E. coli* have shown that exposure to HOCl can deplete bacterial antioxidant enzymes, including SOD and glucose-6-phosphate dehydrogenase (G6PD), which potentiates oxidative damage caused by ROS when cells enter an aerobic environment [77].

Oxidative stress induced by ROS can lead to covalent modifications of amino acids, affecting protein structure, including carbonylation of amino acid side chains of proline, arginine, lysine, and threonine, which is irreversible. Carbonyl derivates can also be formed in histidine, lysine, and cysteine as a result of reactions with secondary oxidation products, including carbonyl compounds on carbohydrates and lipids and advanced glycation/lipoxidation end products [78]. Notably, side chains of methionine and cysteine are susceptible to oxidation due to an electron-rich sulfur atom [41].

**Figure 2:**
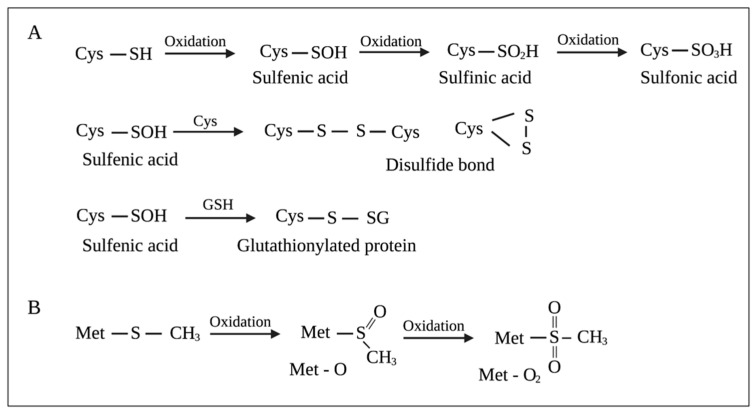
Oxidation of (**A**) cysteine (Cys) and (**B**) methionine (Met) residues on proteins by reactive oxygen species (ROS). GSH: glutathione, Met-O: Methionine sulfoxide, Met-O_2_: methionine sulfone. This figure was created with BioRender.com (accessed on 10 September 2021).

The cysteine (Cys) side chain bears a thiol (–SH) functional group, making it a strong nucleophile. Free Cys has a pK_a_ of approximately 8.6, which can be decreased by 3–4 units in the presence of strong positively charged residues, favoring the deprotonation of thiol (–SH) and the formation of thiolate (–S^−^) at physiological pH. Thiolates rapidly react with electrophiles and oxidants. Two-electron oxidants, such as H_2_O_2_, react with thiolates to produce sulfenic acids (–SOH). One-electron oxidants, O_2_^−^ and HO^•^, react with thiolates to produce thiyl radicals (–S^•^), which can then react with HO^•^ to form –SOH [79]. –SOH can react with Cys residues to either form intra-disulfide or inter-disulfide bonds (-S–S-) or react with glutathione to produce glutathionylated proteins (-S–SG). Additionally, –SOH can be further oxidized to sulfinic acids (–SO_2_H) and sulfonic acids (–SO_3_H) [80] (Figure 2A). The oxidation of the thioether (–SR) side chain of methionine (Met) produces methionine sulfoxide (Met-O) (Figure 2B). Further oxidation of Met-O to methionine sulfone (Met-O_2_) occurs to a much lesser extent [78,81] (Figure 2B).

HOCl can also oxidize proteins, which leads to protein unfolding and irreversible aggregation [82]. Similar to ROS, HOCl most rapidly reacts with Cys and Met in the protein side chains owing to their electron-rich sulfur atom. Moreover, the oxidation of Cys and Met by HOCl and ROS generates the same final products [41]. HOCl can damage membrane proteins involved in energy transduction and transport and cause rapid hydrolysis of ATP [83]. It can also oxidize membrane-bound F1-ATP synthase, leading to the disruption of energy production in bacteria [84]. It can also act on membranes by oxidizing and inhibiting transport proteins or depleting metabolic energy necessary for transport [85].

H_2_O_2_ and HOCl also react with iron centers in microbial enzymes, leading to enzyme inactivation. H_2_O_2_ is also capable of causing Fe^3+^ loss and leading to the release of intracellular Fe^2+^ that can react with H_2_O_2_ to produce highly reactive HO^•^. HOCl can react with the Fe^2+^ itself, which also produces HO^•^ [54]. The Fenton reaction between Fe^2+^ ions and H_2_O_2_ is shown in Figure 1C.

ROS and HOCl can also damage DNA. H_2_O_2_ reaction with the Fe–S cluster in enzymes leads to the release of intracellular iron, which localizes along the phosphodiester backbone of DNA [45]. The reaction of H_2_O_2_ with this unincorporated iron leads to the production of HO^•^ that can oxidize ribose moieties or bases and cause a variety of DNA lesions [40]. Guanine residues appear to be particularly susceptible to oxidation due to low reduction potential, which leads to electron movement from guanine to nearby base radicals [86,87]. Hence, the original site of damage is repaired, while lesioned guanine is converted into mutagenic 8-hydroxyguanine, which can base pair with adenine and escape proofread systems [40,88]. Importantly, since iron is required for the Fenton reaction, higher levels of unincorporated cellular iron should increase DNA damage. Indeed, *E. coli* cells that have disrupted iron metabolism due to mutations in the ferric uptake regulator (*fur*) exhibited higher levels of DNA damage [89]. In *P. aeruginosa*, complete *fur* knockouts have not been obtained due to the essential role of the global-iron regulator [90]. However, *P. aeruginosa* mutants with altered expression of *fur* proteins showed increased sensitivity to oxidative stress [91]. Additionally, it was revealed that oxidative stress from O_2_^−^ causes an increase in the intracellular pool of iron in *E. coli*, leading to DNA damage [92]. HOCl reactions with DNA, RNA, and polynucleotides can yield unstable chloramines that degrade to nitrogen-centered radicals. These radicals were shown to cause single- and double-stranded DNA breaks [93].

**Figure 3:**
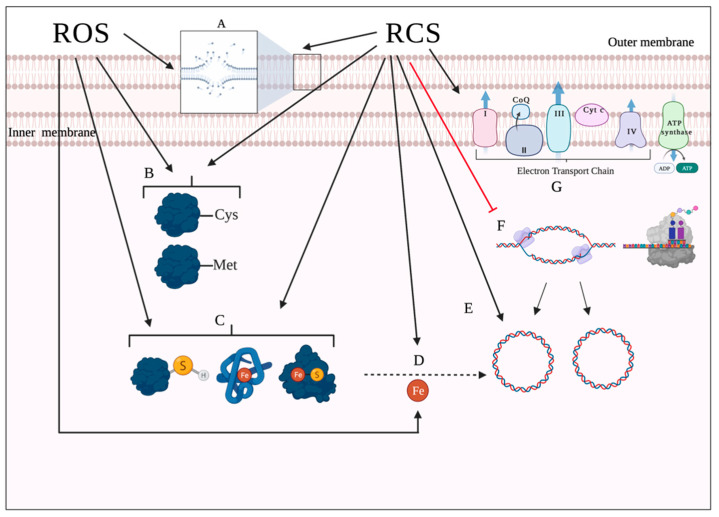
Bacterial targets of reactive oxygen species (ROS) and hypochlorous acid (HOCl), a reactive chlorine species (RCS) with potent antimicrobial effect. (**A**) Lipids represent a common oxidative target for both ROS and HOCl. Both oxidative agents can also induce lipid peroxidation. (**B**) Sulfur-containing side chains of methionine and cysteine are particularly prone to oxidation by ROS and RCS. (**C**) ROS and HOCl actively react with Fe–S clusters and heme proteins in enzymes, which often leads to the release of intracellular iron. (**D**) H_2_O_2_ and HOCl can react with the intracellular iron released after oxidation of Fe–S clusters. This reaction produces HO^•^ that oxidatively damage DNA. (**E**) HOCl can directly damage DNA through deamination, oxidation, and formation of single-stranded breaks and cross-links. (**F**) HOCl can also inhibit DNA and protein synthesis by the inactivation of proteins. (**G**) HOCl can also interfere with the electron transport chain, decreasing energy production. H_2_O_2_: hydrogen peroxide; HO^•^: hydroxyl radicals. This figure was created with BioRender.com (accessed on 10 September 2021).

Lipids represent another target site of oxidative damage. The process of lipid damage is called lipid peroxidation and occurs in three steps [29,94]. In the initiation step, a reactive oxygen metabolite, most likely HO^•^, abstracts a hydrogen atom from a proximal unsaturated lipid (LH), forming a fatty acid radical (L^•^) and triggering a peroxidation chain. In the presence of oxygen, the fatty acid radical (L^•^) will react with oxygen to produce peroxyl radical (LOO^•^) in the propagation stage. The peroxyl radicals (LOO^•^) can, in turn, abstract hydrogen atoms from nearby unsaturated fatty acids, forming lipid hydroperoxide (LOOH) and fatty acid radical (L^•^), hence amplifying the reaction chain [94,95,96]. Additionally, LOO^•^ can decompose into aldehydes, damaging membrane proteins [97]. The last step in the process, chain termination, occurs when LOO^•^ interacts either with an antioxidant or another lipid radical [94]. Importantly, the propagation step in the process requires polyunsaturated fatty acids, implying that saturated and monounsaturated fatty acids can only undergo oxidation [40,94]. HOCl can react with the unsaturated bonds in fatty acids and produce chlorohydrins. These molecules are more polar than unmodified lipids and thus can disrupt membrane integrity, which leads to increased membrane permeability and loss of membrane function [54]. Figure 3 summarizes the bacterial targets of ROS and HOCl.

## 3. Oxidative Stress Responses in *P. aeruginosa*

**Figure 4:**
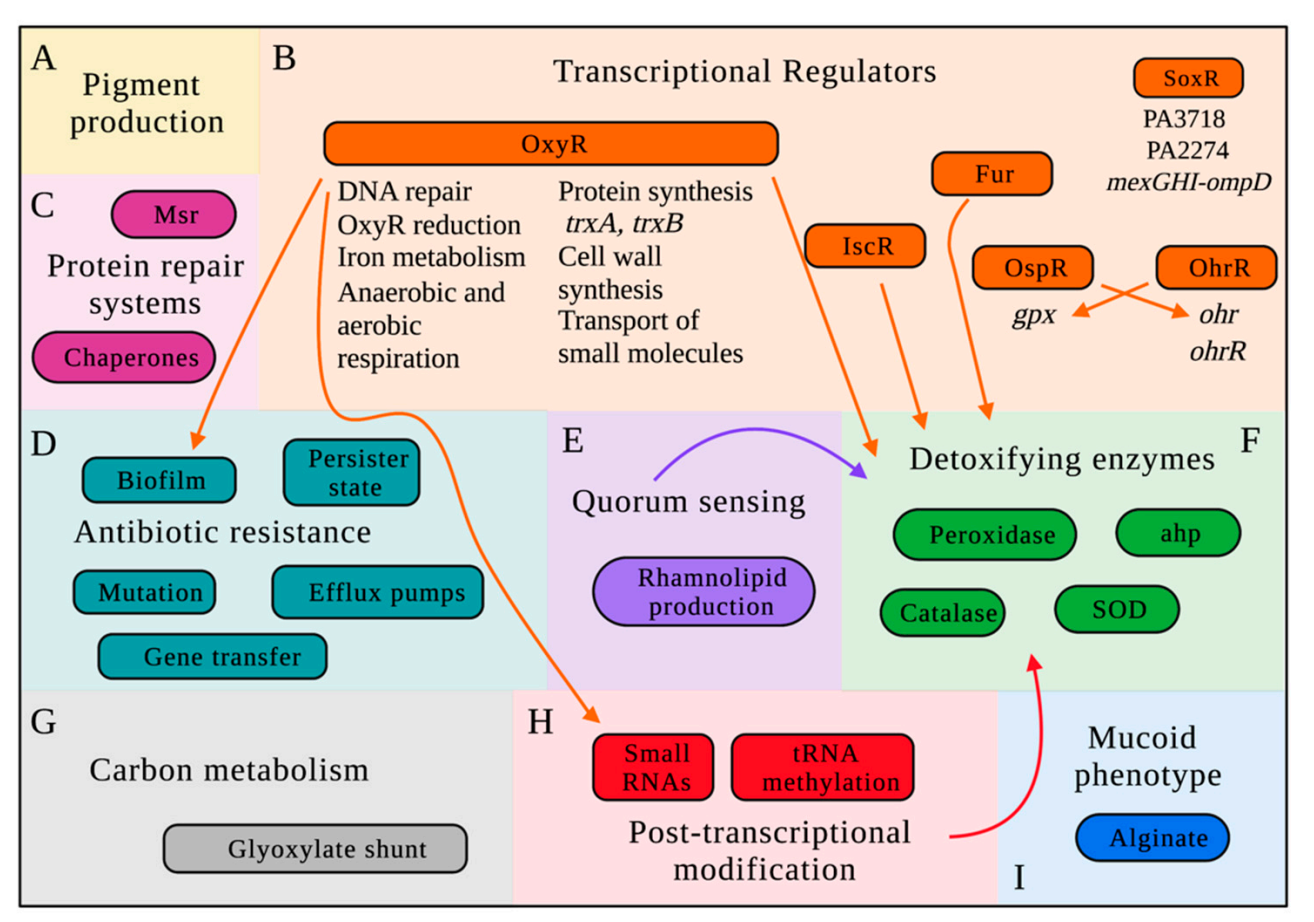
Simplified schematic overview of the interconnected oxidative stress responses adopted by *Pseudomonas aeruginosa*. (**A**) Production of pigments. (**B**) Transcriptional regulators activated by oxidizing agents: OxyR, which has been shown to regulate the expression of several phenotypes, such as the formation of biofilm and induction of small RNAs, in addition to the induction of genes involved in oxidative detoxification (*katB*, *ahpCF*, and *ahpB*); Fur and IscR, which play roles in the expression of detoxifying enzymes; OspR and OhrR, which are homolog regulators that present interconnected activity; and SoxR, which has been shown to activate six genes that are in three transcriptional subunits (PA3718, which encodes an efflux pump of the multiple facilitator superfamily; PA2274, an unknown protein; and four genes in the *mexGHI-ompD* operon). (**C**) Activation of chaperones and the Methionine sulfoxide reductase (Msr) system. (**D**) Antibiotic resistance due to increased mutation frequency, horizontal gene transfer, expression of efflux pumps, and persister state. (**E**) Induction of quorum sensing genes, which are involved in rhamnolipid production and detoxifying enzymes. (**F**) Detoxification of toxic oxygen and chlorine species by antioxidant enzymes, such as catalases (*katA* and *katB*), peroxidases, superoxide dismutase (*sodM* and *sodB*), and alkyl hydroperoxide reductase (*ahpA*, *ahpB*, *ahpCF*, *ohr*). (**G**) Genes involved in carbon metabolism, more specifically in the glyoxylate shunt and the Entner–Doudoroff pathaway were involved in *P. aeruginosa* survival under oxidative stress. (**H**) Methylation and induction of small RNAs. (**I**) Induction of the mucoid phenotype due to the overexpression of alginate. Msr: Methionine sulfoxide reductase; SOD: superoxide dismutase; ahp: alkyl hydroperoxide reductase. This figure was created with BioRender.com (accessed on 10 September 2021).

Microorganisms are constantly surrounded by oxidizing agents, whether produced endogenously by aerobic respiration or exogenously by the host cell. Therefore, almost all bacterial species have developed several adaptive responses to manage the toxic effects of these agents, including changes in the expression of genes encoding detoxifying proteins and enzymes and the activation of transcriptional regulators [98]. Figure 4 shows a schematic of the main stress responses adopted by *P. aeruginosa* against ROS and RCS.

### 3.1. Evaluation of Oxidative Stress Responses of P. aeruginosa by Transcriptomic Studies

Transcriptomic analysis refers to the study of all RNA transcripts produced by the genome of a cell under specific conditions. Through the analysis of gene expression, these genome-wide studies provide insights into the defensive responses adopted by an organism and the possible modes of action of antimicrobial agents [99]. For instance, through the evaluation of gene expression of *P. aeruginosa* exposed to ortho-phenylphenol (OPP), Nde and colleagues (2008) were able to conclude that a mode of action of OPP is through its interaction with amino acid residues, which induces protein synthesis to overcome their shortage in the cell [100]. Not surprisingly, transcriptomic analyses have been used to evaluate changes in gene expression of *P. aeruginosa* exposed to different oxidizing agents.

A common finding in transcriptomic studies of the oxidative stress responses of *P. aeruginosa* is the upregulation of genes involved in protective mechanisms by H_2_O_2_ [101,102,103,104], NaOCl [101,105], peracetic acid [106], HOBr, and HOSCN [105]. Among them are detoxifying enzymes, such as *katB*, PA2826 (glutathione peroxidase), *sodM* and *sodB*, *ahpB* and *ahpF*; DNA repair systems, including *recN*, *prtN*, *prtR*; heat shock proteins, such as *ibpA*, *clpB*, and *hscB*; and bacterial virulence *exoS* and *exoT* genes [101,102,103,104,105,106].

Exposure to NaOCl also induced the expression of genes involved in the transport of sulfur-containing compounds, such as taurine, sulfate, and sulfonate [101,107]. It is consistent with prior studies that showed that due to the reaction of HOCl with sulfur-containing molecules in the cells, the transport of these molecules should be induced to compensate their concentration decreases in the intracellular environment [32].

Another common finding of transcriptomic studies is the downregulation of primary metabolic processes genes in *P. aeruginosa* cells exposed to peracetic acid [106] and H_2_O_2_ [102,103,104]. These genes include energy metabolism (*nuo*, *atp*, *coxB*, *coIII*, *ccoP2*, *ccoN2*, *fdxA*, *hcnA-C*, and *eutB*), ribosomal biogenesis (*rps* and *rpm*), purine and pyrimidine synthesis (*pur*, *pyr*), fatty acid synthesis (*accD*, *accC*, *fabABF1*), polyamine and uptake genes (*speADE and potABCD* operon), and the *sec*-dependent protein translocation pathway (*secABDEFGY*) [102,103,104,106]. Furthermore, NaOCl exposure downregulated genes involved in carbon metabolism, including the Entner–Doudoroff (ED) and Embden–Meyerhof–Parnas (EMP) pathways and the transport of hexose molecules [101,107]. These results suggest that oxidizing agents provoke a decrease in energy production and metabolism, which could favor the survival of these cells under harsh conditions.

The studies conducted by Palma et al. (2004) and Salunkhe et al., 2005 showed that the exposure of *P. aeruginosa* to 1 and 10 mM of H_2_O_2_, respectively, induced the expression of several genes involved in iron homeostasis. On the other hand, Chang et al. (2005) reported the repression of iron genes regulated by the ferric uptake regulator (Fur) (e.g., *pvdS*, *fpvA*, and *fptA*) [102,103,104]. Iron is important for several cellular processes, including enzymatic activity, the expression of virulence factors, the transport of molecules, and the activation and decomposition of peroxides [108]. The discrepancy in these results could be attributed to the differences in the experimental conditions employed. For instance, Salunkhe et al. (2005) treated *P. aeruginosa* cells in the stationary phase, while Palma et al. (2004) used cells in the log phase. Therefore, the upregulation of iron-related genes could be an initial adaptation of the cells to H_2_O_2_ stress [103]. Moreover, Chang et al. (2005) reported an important finding was the overexpression of the F-, R-, and S-type pyocin induced by H_2_O_2_ exposure as a consequence of DNA damage [104].

In another study, Small and colleagues (2007) compared the gene expression profile of *P. aeruginosa* PAO1 upon exposure to H_2_O_2_, NaOCl, and peracetic acid [107]. The authors showed that the expression of a higher number of genes was altered in response to NaOCl treatment, confirming the strong effect of this agent on cellular molecules [32]. Overall, exposure to H_2_O_2_ resulted in the lowest number of genes dysregulated compared to the other oxidants. Peracetic acid strongly upregulated the genes *glpK* and *glpD* (glycerol-3-phosphatase and glycerol-3-phosphate dehydrogenase, respectively), which suggests that under peracetic acid stress, the cells use glycerol and glycerol-3-phosphate as the substrate for growth. Lastly, H_2_O_2_ strongly induced cellular protection genes compared to the other agents [107].

Farrant et al. (2020) evaluated the transcriptional profile of PA14 after exposure to 2.2 mM of NaOCl and 0.8 mM of HOSCN. They found that 70% of the genes upregulated by HOSCN were also induced by NaOCl, indicating that different agents share many bacterial stress responses. These genes belong to the noncoding RNA, protein secretion/export apparatus, and antibiotic resistance and susceptibility [109]. In the study conducted by Groitl et al. (2017), the authors analyzed gene expression changes of *P. aeruginosa* PA14 treated with HOCl, HOBr, and HOSCN. In addition to the induction of protective enzymes by all three oxidants, the regulator MexT involved in bacterial virulence was also induced by all three treatments, and the regulators Fur and PchR were induced by only HOCl. Another important result obtained by these authors was that HOCl and HOBr upregulated several chaperones and heat shock proteins (e.g., *dnaK*, *ibpA*, *groES*, *hskU/V*), while HOSCN induced only two chaperones (*hslV* and *grpE*). Moreover, HOCl and HOBr also induced several genes related to motility and attachment, chemotaxis, and nucleotide biosynthesis compared to HOSCN. On the other hand, HOSCN induced a higher number of genes in antibiotic resistance or membrane proteins, indicating that the primary mode of action of this agent is its membrane structures [105].

Transcriptomic studies and their similarities provide insights into the adaptive responses adopted by bacteria under oxidative stress. Genes that are mostly regulated in different studies and experimental conditions probably play important roles in bacterial survival under oxidative stress conditions. For instance, most studies revealed the upregulation of detoxifying enzymes, such as catalase and superoxide dismutase, which are well known to be an important protective strategy adopted by bacterial cells to manage oxidative damage. Furthermore, these studies could also help understand bacterial pathogenesis during infection. For example, the induction of genes related to bacterial virulence by oxidative stress could help understand and reduce bacterial pathogenicity during lung infections in CF patients. Overall, the differences obtained among the studies are attributed to the experimental conditions employed, such as the concentration of oxidizing agents, the bacterial strains utilized, the growth phase of the cells during the treatment, and the treatment time.

### 3.2. Cellular Responses to Oxidative Stress

#### 3.2.1. Antioxidant Enzymes

The removal of oxidizing agents is the primary action taken by cells exposed to oxidative stress [110]. The detoxification of toxic oxygen species is done by specialized enzymes, such as catalase, superoxide dismutase (SOD), peroxidase, and alkyl hydroperoxidase, which catalyze the conversion of these species into less toxic substances. These enzymes are essential for the survival of bacteria under oxidative stress.

SODs are periplasmic enzymes that catalyze the conversion of O_2_^−^ into H_2_O_2_, while catalases and peroxidases are present in the cytoplasm and generate H_2_O and O_2_ from H_2_O_2_. The removal of toxic oxygen species is connected to the action of these enzymes, in which SODs are considered the first defense involved in superoxide detoxification [111]. *P. aeruginosa* has two SODs: one that is Fe-cofactored (Fe-SOD; SodB), which is found in all growth conditions, and another one that uses manganese (Mn-SOD; SodM, previously called SodA) as a cofactor, which has been shown to be induced under iron starvation and alginate production [112,113]. Compared to Mn-SOD, Fe-SOD presents a more pronounced effect in *P. aeruginosa* growth under aerobic conditions, protection against superoxides, and pyocyanin production [112,114]. A significant reduction in catalase production was detected in Fe-SOD mutants compared to Mn-SOD, supporting the roles of this enzyme in oxidative stress protection [114]. Furthermore, Fe-SOD also contributes to the long-term survival of *P. aeruginosa* within human and murine macrophages, inhibits the activation of autophagy [111], and is involved in the *P. aeruginosa* infection process of a *Bombyx mori* model [115].

*P. aeruginosa* has three catalases (*katA*, *katB*, and *katC*) and four alkyl hydroperoxide reductases (*ahpA*, *ahpB*, *ahpCF*, and *ohr*) that detoxify H_2_O_2_. Of these, *katA* is considered the major catalase of *P. aeruginosa*, and its expression is controlled by several systems (e.g., quorum sensing (QS), iron levels, anaerobic regulator (ANR), OxyR, and IscR) [116,117]. It is extremely stable and is expressed continuously throughout all bacterial growth phases, with an observed overexpression detected during the stationary phase. In addition to its antioxidant activity, it is also involved in *P. aeruginosa* virulence in a *Drosophila melanogaster* and mice infection models, in which *katA* mutants presented reduced killing-effects on infected animals compared to the wild-type [118]. On the other hand, the expression of *katB* is induced by H_2_O_2_ stress [117,119]. KatC (also called KatE due to its homology with *E. coli* catalase KatE) is a temperature-inducible catalase that requires the formation of a disulfide bond for its activity [120]. The role of KatC in cellular processes and protective mechanisms of *P. aeruginosa* have not been completely investigated [121]. Catalase is also involved in the protection against the H_2_O_2_ of *P. aeruginosa* biofilms by inhibiting the penetration of this oxidizing agent into this structure [119,122,123] and contributing to *P. aeruginosa*’s resistance in chronic infections [123].

Alkyl hydroperoxide reductase generates alcohol and water from H_2_O_2_ and organic hydroperoxide. Similar to *katA*, *ahpA* is involved in the oxidative stress response against endogenously produced ROS since it is continually expressed during all phases of aerobic growth. Conversely, AhpCF is overexpressed under exogenous oxidative stress, being considered a protective protein against the damage caused by oxidizing agents [124,125]. In addition to its detoxifying effect, AhpCF has also been shown to possesses chaperone activity [126,127].

#### 3.2.2. Protein Repair Systems

Methionine sulfoxide reductases (Msr) are enzymes that catalyze the reduction of Met-O generated under oxidative conditions to Met. It is conserved and found in several organisms. Most bacterial species, including *P. aeruginosa*, have two Msr, *msrA* and *msrB* [128,129]. The oxidation of Met generates two isomers of Met-O (i.e., Met-(S)-O and Met-(R)-O). MsrA specifically reduces Met-(S)-O, and MrsB reduces Met-(R)-SO [130,131]. In addition to its antioxidant effect, Msr is also involved in the pathogenesis of several bacterial species (reviewed in [130]). In *P. aeruginosa*, the level of MsrA is kept constant throughout several growth phases under physiological conditions, suggesting that this enzyme is ready to detoxify oxidized Met when the cells are exposed to oxidative stress. On the other hand, the expression of *msrB* is induced under oxidative stress, such as NaOCl exposure. In this context, *msrA*, *msrB*, and *msrA msrB* double mutants are significantly more susceptible to NaOCl and H_2_O_2_ than the wild-type. In addition to the oxidative protection, these mutants also presented reduced virulence in a *Drosophila melanogaster* model [129].

Oxidizing agents, such as HOCl, HOBr, and HOSCN, cause significant protein unfolding, which leads to unspecific protein aggregation [53,105]. Therefore, it is not surprising that bacteria have established diverse response mechanisms against the proteome damage caused by these agents. In this context, the major response against protein unfolding and aggregation is the activation of chaperones. The activation of these proteins by RCS stress in several bacterial species was reviewed in [32,53,132]. For instance, a study conducted by Groitl et al. (2017) showed that the overproduction of polyP by *P. aeruginosa* cells under HOCl stress and the substantial protein aggregation when this molecule is absent demonstrates the roles of chaperones in bacterial survival under RCS stress [105]. The roles of polyP as a chaperone have also been described in different studies for different Gram-negative species (reviewed in [32]).

#### 3.2.3. Quorum Sensing (QS)

Quorum sensing (QS) is a mechanism used by bacteria to communicate with each other in response to cell density [133]. Most of the virulence factors produced by *P. aeruginosa*, including biofilm formation, motility, antibiotic resistance, the response to host immune system, and the secretion of proteases, iron chelators, and efflux pumps, are controlled at least in part by the QS system [134,135]. Furthermore, QS has also been shown to play important roles in the oxidative stress survival of *P. aeruginosa.* For instance, it regulates rhamnolipid production, known to be involved in oxidative stress responses due to foam formation on the surface of media. This foam prevents the entry of oxygen and, therefore, is considered a protective substance against toxic oxygen species [136,137].

The QS system regulates cell-to-cell communication through the interaction of QS signal molecules (N-acyl-L-homoserine lactones) with transcriptional regulators, which induces the expression of target genes. *P. aeruginosa* has three major and well-characterized QS systems: las, rhl, and the *Pseudomonas* quinolone signal (PQS) [138]. The Las system is composed of a transcriptional regulator LasR and the signal molecule N-(3-oxododecanoyl)-L-homoserine lactone (3-oxo-C_12_HSL), synthesized by the enzyme LasI. Likewise, the rhl system is constituted of the transcriptional regulator RhlR and the signal molecule N-butanoyl-L-homoserine lactone (C_4_-HSL), synthesized by RhlI synthase. The PQS system is composed of the transcriptional regulator PqsR and the signal molecule 2-heptyl-3-hydroxy-4-quinolone [139,140]. The las system controls the activation of the rhl and PQS systems hierarchically in response to an increase in cell density. An increase in cell population stimulates the synthesis of 3-oxo-C_12_HSL, which forms a complex with the transcriptional regulator LasR, activating the transcription of the genes lasI and rhlR [141]. PqsR (MvfR) was also shown to exert direct control on LasR and RhlR during early and mid-exponential phases, suggesting an interconnected regulation of these three systems [134].

In addition to its effects on *P. aeruginosa*’s virulence and pathogenicity, the roles of QS in bacterial survival under oxidative stress have also been demonstrated in several studies. QS controls the expression of antioxidant enzymes [142], and the presence of SodM was not detected in *lasI* and *lasR* mutants [143]. The impaired production of detoxifying enzymes by QS mutants explains their susceptibility to oxidizing agents. Furthermore, only cells with functioning QS systems can survive and effectively respond to oxidative stress [144]. The response of LasR to oxidative stress was shown to be due to the formation of a disulfide bond between two cysteine residues in the molecule (Cys201 and Cys203) [145].

Häussler and Becker (2008) showed that *pqs* mutants were more tolerant to H_2_O_2_ than the wild-type, indicating that PQS induces susceptibility of *P. aeruginosa* to oxidative stress. PQS also decreases the amount of intracellular ROS and exhibits a potent antioxidant effect due to its electron-donating potential and by inducing undamaged bacteria to enter a low metabolic state and, consequently, to become less susceptible. It also has a pro-oxidant effect, in which it also generates ROS through the Fenton reaction [146]. PqsR regulates the key genes of type 6 and type 2 secretion systems (T6SS and T2SS, respectively) at early stages of growth and induces the oxidative stress genes *ahpC-ahpF*, *trxB2-ahpB*, and *dps*, in which these mutants were less tolerant to H_2_O_2_ than the wild-type, demonstrating its roles in oxidative stress. Furthermore, this oxidative stress effect provoked higher resistance to the ß-lactam Meropenem than the wild-type [134].

#### 3.2.4. Induction of a Mucoid Phenotype

The colonization of the lungs of CF patients by *P. aeruginosa* is the leading cause of morbidity and mortality among these individuals [147]. In the earlier stages of infection, *P. aeruginosa* appears as a nonmucoid bacterium; however, it switches to a mucoid phenotype in the later phases of colonization [148]. The conversion of a nonmucoid to a mucoid phenotype is caused by an overproduction of a capsule composed mainly of the exopolysaccharide alginate and leads to high mortality rates, poor prognosis, and persistent infection in CF patients [23]. Alginate is composed of two uronic acids (i.e., β-D-mannuronic acid and α-L-guluronic acid) linked together through glycosidic bonds [24] and is involved in biofilm formation, bacterial adherence to surfaces, scavenging of ROS, and physical protection against antibiotics, oxidizing agents, and the immune system [149,150,151].

The biosynthesis of alginate is a complex process that involves several genes. It is regulated by the sigma factor σ^22^ (AlgU, also called AlgT), which is controlled by the MucABCD system [152]. The overproduction of alginate and the consequent conversion to a mucoid phenotype is often due to a mutation in the gene that encodes the anti-σ factor MucA, but it can also occur by the degradation of MucA by the AlgW protease [153,154]. MucA is a transmembrane protein that binds to the alternative sigma factor AlgU, inhibiting the expression of the alginate operon [148,149]. AlgU, in turn, is required for the expression of *algD*, which is a gene encoding for a GDP-mannose 6-dehydrogenase and is the first gene of the alginate biosynthesis operon and therefore essential for the production of alginate [151].

The conversion to a mucoid phenotype has been described as an adaptive response to toxic ROS. Oxidizing agents, such as H_2_O_2_, can induce the overproduction of alginate in nonmucoid *P. aeruginosa* cells and consequently promote the conversion to the mucoid phenotype [136]. Mathee and colleagues (1999) showed that non-mucoid *P. aeruginosa* PAO1 grown in biofilms presented the mucoid phenotype and overproduced alginate after exposure to sublethal concentrations of H_2_O_2_ (1.0 mM or 2.5 mM) and human polymorphonuclear leukocytes (PMNs), while this phenomenon was not observed in untreated biofilms. It indicates that the overproduction of alginate in *P. aeruginosa* biofilms is an adaptive response to the immune system and its toxic oxygen species [154]. Other studies have reported that genes involved in alginate biosynthesis pathways are affected by H_2_O_2_ and paraquat exposure. For instance, *algU* mutants were more sensitive to paraquat [151] and hypochlorite [155] than the wild-type, and the mutations of *algR* impaired the stress response of *P. aeruginosa* to H_2_O_2_ [156].

Boucher and colleagues (1996) demonstrated the roles of the genes *algW* and *mucD* on *P. aeruginosa* survival under H_2_O_2_ and paraquat stress and their roles in the conversion to the mucoid phenotype. The authors showed that the inactivation of *algW* increased the sensitivity of *P. aeruginosa* PAO1 to H_2_O_2_ and paraquat, and mutations on the *mucD* gene conferred sensitivity to only H_2_O_2_ [157]. The difference between the protection levels conferred by these genes against H_2_O_2_ and paraquat on the cells can be explained by the fact that paraquat acts mainly in the cytoplasm, while MucD is a periplasmic protein [150,157]. Moreover, a mutation in the genes *algW* and *mucD* induced the conversion to the mucoid phenotype since they act as negative regulators of alginate synthesis [157].

#### 3.2.5. Pigment Production

*P. aeruginosa* secretes several types of extracellular pigments, of which phenazines are the most common. Many *P. aeruginosa* strains also produce pyocyanin, pyoverdine, pyorubin, and pyomelanin. These pigments are involved in bacterial virulence and survival [158,159]. Among the different types of phenazines secreted by this bacterium, pyocyanin, a blue-green substance, is produced by most *P. aeruginosa* isolates [160]. It is considered an important virulence factor of *P. aeruginosa* and plays a vital role in iron metabolism and antimicrobial effect against competitors [161]. Pyoverdine is a fluorescent yellow-green siderophore involved in iron uptake [162], while pyomelanin and pyorubin are non-fluorescent brown and red-brown pigments, respectively [161].

The protective effect of *P. aeruginosa* pigments against oxidative stress induced by paraquat [159], photodynamic therapy [158], and H_2_O_2_ [163] have been reported. The exposure of *P. aeruginosa* to paraquat increased the growth and production of a dark-brown melanin-like pigment in a *P. aeruginosa* dark-brown hyperpigmented (HP) strain, suggesting that this dark-brown pigment helped the bacteria to survive under oxidative conditions and might be an oxidative response against superoxide. This hypothesis was confirmed when this pigment, which contains several antioxidant molecules, was added to *E. coli* cells treated with paraquat, and it protected the cells against the oxidative effect of this superoxide generating agent [159]. The production of pyomelanin is induced by the inactivation of the gene *hmgA.* The disruption of this gene increased the persistence of the bacteria during lung infection and oxidative tolerance but did not promote tolerance to antibiotics. Furthermore, the supernatant of the hyperproducer mutants protected the wild-type against the effects of H_2_O_2_ [163]. Together, these results suggest that the pigments produced by *P. aeruginosa* cells also contribute to their protection against harsh environmental conditions created by the presence of oxidizing agents, probably by containing detoxifying enzymes.

In addition to the protective effect of *P. aeruginosa* pigments, some of them are considered toxic to the cells. For instance, pyocyanin generates superoxide and H_2_O_2_ by reducing molecular oxygen, which can cause irreversible oxidative damage to the host cells and the bacteria. Therefore, *P. aeruginosa* adopts several mechanisms to counteract the auto-poisoning effects of pyocyanin, such as inducing the formation of biofilms and decreasing energy production [164], overexpressing antioxidant enzymes, such as catalase and SOD, and blocking the uptake of this phenazine [165]. Meirelles et al. (2018) demonstrated that exposure to pyocyanin strongly induced H_2_O_2_ sensing genes regulated by the OxyR transcriptional regulator, including *ahpC*, *ahpB*, *ahpF*, thioredoxin reductase (*trxB2* and *trxA*), and *katB*. Moreover, pyocyanin also significantly upregulated genes involved in the efflux system (i.e., *mexG* and *mexH* of the efflux system *mexGHI-ompD* and *mexEF-oprN*), and slightly regulated DNA repair genes (i.e., *recA* and *lexA*). Furthermore, three genes belonging to the *isc* operon (i.e., *iscA*, *iscU*, and *fdx2*) involved in Fe–S cluster biosynthesis were upregulated in the presence of pyocyanin, which is related to the oxidative damage caused by pyocyanin to sulfur-containing compounds. Previous exposure to pyocyanin also induced the cells to enter a persister state when cultured in a nutrient-depleted media. However, when the cells were transferred to nutrient-rich media, they became sensitive again to pyocyanin, indicating that the toxicity of pyocyanin depends on environmental conditions [164]. The production of pyocyanin by *P. aeruginosa* also induced the persister state in *A. baumannii* [166].

#### 3.2.6. Carbon Metabolism

According to the growth conditions, *P. aeruginosa* can adapt and utilize distinct pathways to metabolize different carbon sources, which provides it with the ability to survive in different environmental conditions [167]. For instance, *P. aeruginosa* PAO1 and other pseudomonads catabolize glucose predominately through the Entner–Doudoroff pathway (EDP) [168], which could be considered a strategy to counteract oxidative stress since this pathway is extremely efficient in generating the NADPH required for the function of several antioxidant responses [168,169].

In addition to providing adaptation to several environmental conditions, studies have shown that other carbon metabolism pathways of *P. aeruginosa*, such as the glyoxylate shunt (GS), also contribute to bacterial survival under oxidative stress [170,171]. GS is a two-step metabolic route composed of two enzymes (i.e., isocitrate lyase (*aceA*) and malate synthase (*glcB*)) and is an alternative to the tricarboxylic acid cycle (TCA). In this pathway, aerobic bacteria metabolize acetate and fatty acids as carbon sources [172]. An example of this kind of environment is the CF sputum, where the concentration of fatty acid is exceptionally high [171].

The *P. aeruginosa* and *E. coli* isocitrate lyase have 27% identity, while *P. aeruginosa* malate synthase shares 59% sequence identity with *E. coli* malate synthase. In addition to being upregulated by the excess of fatty acid and acetate, *aceA* expression is induced under H_2_O_2_ exposure and iron limitation. At the same time, *glcB* is overproduced by redox-active compounds and antibiotics exposure. GS was shown to regulate the intracellular amount of iron, and the susceptibility of GS mutants to oxidative stress could be due to the interaction of H_2_O_2_ with intracellular iron [171]. Ahn and colleagues (2017) showed that *aceA* is activated in response to oxidative stress, in which the overproduction of the efflux pump *mexE* could contribute to the bacterial response to these toxic agents. Furthermore, increased EPS production, biofilm formation, and pyoverdine and pyocyanin synthesis were observed in the *aceA* mutant [170].

Although the molecular mechanisms behind their roles in the oxidative stress response have not been completely elucidated, other proteins involved in carbon metabolism have also been described to be involved in bacterial survival under oxidizing conditions. For instance, enolase, an enzyme involved in carbon metabolism and an important component of the *E. coli* RNA degradosome [173], was shown to possesses roles in *P. aeruginosa* growth and virulence in a murine acute pneumonia model. It was also involved in oxidative stress tolerance by affecting the production of *ahpB* and *ahpC* in an OxyR-independent manner [174]. In another study, the authors showed that Crc, a regulator that controls carbon catabolite, participates in the oxidative stress response in *P. aeruginosa* by controlling the metabolic flux through NADP^+^-dependent dehydrogenases and maintaining the *P. aeruginosa* metabolism [175].

#### 3.2.7. Post-Transcriptional Modification

Several RNA transcripts undergo post-transcriptional modification to become a mature and functional molecule, contributing to a more effective translation of RNA into protein [176]. Studies have reported the roles of tRNA methylation, RNases, and small RNAs on oxidative stress response genes. The upregulation of *trmB*, a gene that encodes a tRNA guanine-N7-methyltransferase that catalyzes 7-Methylguanosine (m^7^G) modification in tRNA, increases the amounts of m^7^G modified tRNA, which improves the translation efficiency of Phe and Asp enriched mRNAs, such as *katA* and *katB* [177]. Jaroensuk and colleagues (2016) also showed that the methyltransferase TrmJ also plays a role in oxidative stress response by regulating the expression of *oxyR-recG*, *katB*, and *katE* [178].

The endoribonuclease YbeY is involved in ribosome quality control and maturation and post-transcriptional regulation of gene expression [179]. It is also required for bacterial survival in a murine acute pneumonia model. This endoribonuclease has also been shown to play important roles in the oxidative stress response. A mutation in the *P. aeruginosa yebY* gene reduced the expression of *katA* and *katB*, and this mutant also presented higher sensitivity to neutrophiles exposure than the wild-type. YbeY controls the expression of *katA* through the sigma factor RpoS, in which YbeY affects the *rpoS* translation by upregulating ReaL, a small RNA (sRNA) that represses *rpoS* translation. Furthermore, YebY interacts with RpoS and promotes its degradation [180]. The roles of sRNAs on oxidative stress were also shown by Gómez-Lozano et al. (2014). In this study, the authors demonstrated that several antisense small RNAs were differentially expressed under different experimental conditions, including osmotic pressure, oxidative stress, and antibiotic treatment, suggesting that they may play regulatory roles in *P. aeruginosa cells* [181].

#### 3.2.8. Other Stress Response Mechanisms

As the study of oxidative stress responses adopted by bacteria progresses, several proteins known to be involved in specific pathways have also been recognized for being employed by bacteria as a survival strategy. In *P. aeruginosa*, several findings have been made in this direction. For instance, studies have shown that lipotoxin F (*lptF*) is involved in the oxidative stress response and adhesion to human lung epithelial cells, and its expression is stimulated by the conversion to the mucoid phenotype [155]. Furthermore, acyl carrier proteins [182], flavohemoprotein [183], PppA-PpkA kinase [184], flavodoxins [185], and the *phnW* pyruvate aminotransferase [186], which are involved in fatty acid biosynthesis, nitric-oxide detoxification, protein phosphorylation, electron transfer, and phosphonate degradation, respectively, have also been shown to play important roles in adaptive response and *P. aeruginosa* survival under oxidizing conditions.

Oxidative stress has also been shown to induce other phenotypic changes in *P. aeruginosa*, mainly in chronic infection where the immune system and therapeutic agents impose adverse conditions to the survival of pathogens [187]. In this context, resistance to antibiotics, biofilm formation, and mucoid and hypermutable phenotypes play important roles in *P. aeruginosa* survival under oxidizing environments [187,188]. For instance, the presence of hypermutable *P. aeruginosa* in the lungs of CF patients was reported to be associated with antibiotic resistance and increased time of infection. This hypermutable phenotype was provoked by DNA mutations induced by oxidizing agents [189]. Furthermore, double-strand breaks caused by oxidative stress in *P. aeruginosa* biofilms were shown to induce several DNA repair systems, generating mutations that promote the high adaptability and diversity of these communities [190].

### 3.3. Transcriptional Regulators Involved in P. aeruginosa Oxidative Stress Responses

#### 3.3.1. OxyR: The Master Regulator of Oxidative Stress

OxyR is a 34 kDa H_2_O_2_-sensing transcriptional regulator that belongs to the LTTR family (LysR Type Transcriptional Regulators). It is composed of four different domains (i.e., DNA binding domain, H_2_O_2_ sensing, transactivation, and tetramerization) [191] and is commonly found in almost all Gram-negative bacteria [192,193,194]. It is also present in some Gram-positive species, such as *Streptomyces avermitilis* [195] and *Staphylococcus aureus* [196]. OxyR is the central, most frequently studied, and the best-characterized transcriptional regulator involved in oxidative stress responses by the induction of the transcription of protective genes, such as catalases and alkyl hydroperoxides [197]. In *P. aeruginosa*, most of the genes involved in response to H_2_O_2_ and organic peroxides are controlled by OxyR [192].

The activation of OxyR in *P. aeruginosa* is done by the oxidation of Cys199 and Cys208. Furthermore, a third cysteine residue (Cys296) might also be involved in OxyR activation. This cysteine residue is not conserved in *E. coli* and is found only in a few beta-proteobacteria. In addition, *P. aeruginosa* OxyR presents positive and negative regulation effects [191]. For instance, *katA* is positively and negatively regulated by OxyR, while *katB* is positively regulated [117].

In *P. aeruginosa*, a 34kDa OxyR transcriptional regulator induces the expression of one catalase and two alkyl hydroperoxide reductase (i.e., *katB*, *ahpB*, and *ahpCF*, respectively). It also plays important roles in DNA repair since it is located in an operon with the DNA helicase RecG [125]. In addition to the protection to H_2_O_2_, the *oxyR-recG* operon is also involved in *P. aeruginosa* protection against paraquat [125] and HOCl [109].

Recent studies have shown that *P. aeruginosa* OxyR regulates numerous genes involved in stress responses under oxidative stress. Wei and collaborators (2012) demonstrated that OxyR also regulates ~122 genes in response to H_2_O_2_ stress [192]. It binds to the promoter of several genes and regulates the expression of those involved in H_2_O_2_ detoxification (e.g., *katA*, *katB*, *ahpB*, and *ahpCF*), iron metabolism (e.g., *dps* and *pvdS*), biofilm formation (e.g., *pf4* and *bdlA*), protein synthesis (e.g., *rpsL*, *cca*, *aspS* and *alsS*), cell wall synthesis (e.g., *lpxC*), QS (e.g., *rsaL*), aerobic and anaerobic respiration (e.g., *snr1* and *cyoA*), the transport of small molecules (e.g., PA1541 and PA0185), OxyR reduction (e.g., *trxA* and *trxB*), and other regulatory genes (e.g., sRNA). Overall, OxyR represses protein synthesis and oxidative respiration and reduces the expression of iron uptake genes, indicating that bacteria reduce their metabolic activity as a response to oxidative stress. On the other hand, it induces detoxifying defenses, biofilm formation, and the production of sulfur-containing molecules. Detoxifying enzymes and biofilm formation are known to be an important protection for bacterial cells under oxidative conditions. Furthermore, these authors showed that *P. aeruginosa* OxyR binds to the promoter region of a thioredoxin system (*trxA* and *trxB2*), inducing its expression and at the same time also reduces the oxidized OxyR when NAD(P)H is available, indicating an auto-regulatory mechanism [192].

*P. aeruginosa* OxyR was also shown to affect other phenotypes. For instance, OxyR mutants were unable to swarm on agar plates due to the deficiency in rhamnolipids production. They also presented an increased pyocyanin production, suggesting the roles of this transcriptional regulator in bacterial virulence [198]. In addition to that, the inactivation of the *P. aeruginosa* OxyR abolished the utilization of pyoverdine, impairing iron acquisition by the cells [199]. These studies show that OxyR is a regulator that plays an essential role in bacterial survival and adaptation by regulating several genes involved in the detoxification of toxic oxygen species, DNA repair, resistance, and virulence.

#### 3.3.2. OhrR and OspR

OhrR (organic peroxide-sensing repressor) is a transcriptional repressor widely found in Gram-positive and Gram-negative bacteria that belongs to the MarR family and controls the transcription of *ohr* (organic hydroperoxide resistance) [200,201]. *P. aeruginosa* OhrR acts as a repressor of the expression of *ohr* and *ohrR* by biding to their promoter regions. Under oxidative conditions, the formation of a disulfide bond between Cys19, the sensing cysteine, and Cys121 promotes a conformational change in the structure of OhrR, which provokes its dissociation from the promoter and allows the binding of RNA polymerase [202].

Studies have shown the protective effect of OhrR in *P. aeruginosa* under organic hydroperoxide conditions [202,203]. A *P. aeruginosa ohr* mutant was susceptible to *t*-butyl hydroperoxide and cumene hydroperoxide but not to paraquat and H_2_O_2_, suggesting that Ohr is a protective protein against organic hydroperoxide. Although the function of Ohr is similar to the function of AhpC-AhpF, its expression is OxyR-independent [204].

OspR (oxidative stress response and pigment production regulator) is a transcriptional regulator homolog to *P. aeruginosa* OhrR (46.5% identity) [203]. *P. aeruginosa* OspR is part of the oxidative stress response in this bacterium by the production of a glutathione peroxidase (*gpx)*, which is a protective protein that decomposes peroxides using glutathione [205]. *OspR* and *gpx* are strongly induced by organic and lipid hydroperoxides and confer resistance to these compounds and H_2_O_2_ [203].

The effect of OspR occurs by its binding to the promoter region of the *ospR-gpx* operon, which consequently represses the transcription of *gpx* and *ospR.* In addition to its roles in oxidative protection of *P. aeruginosa* cells, OspR is also involved in several cellular pathways, including pigment production, QS, resistance to β-lactam antibiotics in a gpx-independent manner, and virulence in mice [205].

OspR and OhrR are two organic hydroperoxide regulators in *P. aeruginosa* that belong to the two-cysteine subfamily of the OhrR regulators. Similar to OhrR that has a sensing cysteine (Cys19), which plays essential roles in sensing oxidative stress, Cys24 plays essential roles in the protective effect of OspR [203,205]. Atichartpongkul and colleagues (2016) showed that OspR and OhrR have overlapping roles in the oxidative defense of *P. aeruginosa*. They demonstrated that OspR binds to the *ohr* promoter under oxidative stress and regulates its transcription, and OhrR binds to the *gpx* promoter, however, with lower affinity. Therefore, the ability of OspR to bind to the two promoters (*ohr* and *gpx*) highlights its broader protective effect and suggests that the oxidative protection conferred by OhrR and OspR is interconnected [203].

#### 3.3.3. IscR

IscR (iron–sulfur cluster) is a transcriptional regulator that belongs to the Rrf2 family and regulates the expression of genes involved in iron–sulfur ([Fe–S]) cluster biosynthesis. [Fe–S] cluster proteins play essential roles in several cellular processes, including respiration, gene regulation, DNA repair, electron storage, and central metabolism [206,207,208]. In *P. aeruginosa* only the *isc* system encoded by the *iscRSUA-hscBA-fdx2-iscX* operon has been identified [110]. IscR recognizes two different binding motifs: type 1, which is recognized by holo-IscR, and type 2, recognized by both holo- and apo-IscR [209].

IscR has been described to be involved in the stress response of *P. aeruginosa* to oxidative stress [109,207,210] and bacterial virulence [210]. *iscR* mutants presented lower KatA, SodA, and SodB production than the wild-type [207,210], and this gene was required for the activity of KatA at the post-translational level [210]. Since the inactivation of *iscR* provoked a reduction in intracellular iron levels, affecting several iron-dependent pathways [110], and these enzymes require iron to function, the depletion of IscR explains their impaired activity and, consequently, the low tolerance against oxidizing agents.

In addition to its roles in protection against oxidative stress, IscR is also induced under different stress conditions that affect the availability of iron and damage the [Fe–S] cluster, such as iron and thiol depletion and high salt. The genes regulated by *iscR* that are important for the oxidative stress response have not been completely described [110]; however, a few of them have been studied recently. For instance, *fprB*, a gene that encodes a ferredoxin NADP(+) reductase, and *nfuA*, which encodes a [Fe–S] scaffolding protein involved in [Fe–S] cluster biosynthesis, have been described to be regulated by *P. aeruginosa* IscR. These proteins were significantly induced by oxidative agents, such as cycling agents, peroxide, and organic hydroperoxides, and the respective mutants showed increased sensitivity to oxidative stress [211,212]. *fprB* is also essential for bacterial survival under osmotic pressure and metal stress [211]. Overall, the susceptibility to oxidative stress observed in these mutants might be associated with the loss of function of the [Fe–S] cluster-containing proteins and transcriptional regulators. In another study, Boonma and collaborators (2017) showed that the Lys-R family transcriptional regulator FinR was activated by NaOCl and paraquat and mediated the expression of *fprA* under these stressful conditions [213]. Together, these results highlight the role of *fprA* in maintaining iron homeostasis and bacterial survival under oxidative stress conditions.

#### 3.3.4. Fur

The Ferric uptake regulator (Fur) represses the expression of iron uptake genes when enough iron is available to the cells. It was shown that, in *P. aeruginosa*, Fur regulates the production of exotoxin A and siderophores [214,215]. In solution, *P. aeruginosa* Fur is a tetramer and forms dimers in the presence of DNA during its activation. The tetramer form of *P. aeruginosa* Fur is inactive and is activated by its dissociation into dimers and metal binding [216].

Fur has been described to positively activate *P. aeruginosa katA* and *sodB* [217]. Furthermore, Pasqua and collaborators (2017) showed that *P. aeruginosa* Fur mutants present impaired growth in solid media due to pyochelin production inhibition. However, the mutants did not show impaired biofilm formation and pathogenicity in a *Galleria mellonella* model, suggesting that Fur is not required for biofilm formation and bacteria pathogenicity in this in vivo model [218].

#### 3.3.5. SoxR

SoxR is a 17kDa transcriptional regulator of the MerR family that controls O_2_^−^ and NO responses. *P. aeruginosa* does not have the transcriptional regulator SoxS [219], which is frequently activated by SoxR and induces the transcription of several genes [220,221,222]. Furthermore, while SoxR and OxyR are often considered the primary sensors that control stress responses under oxidative conditions [223], *P. aeruginosa* SoxR is not essential for the oxidative stress response and antibiotic resistance [224].

Palma et al. (2005) [224] demonstrated that, under paraquat stress, *P. aeruginosa* SoxR activates six genes that are in three transcriptional subunits by binding to their promoter region. However, it was not activated by H_2_O_2_. The three subunits comprise the following genes: (i) PA3718, which encodes an efflux pump of the multiple facilitator superfamily; (ii) PA2274, which is an unknown protein with similarity to the monooxygenase and that has detoxification function; and (iii) four genes in the *mexGHI-ompD* operon, encoding the multidrug efflux pump of the resistance-nodulation-division superfamily [224]. SoxR might also be involved in *P. aeruginosa* virulence through the expression of the MexGHI-OmpD system since this efflux pump plays roles in QS [225,226]. In addition, *P. aeruginosa* SoxR is required for bacterial virulence in mice with pulmonary infection, suggesting the importance of this regulator in *P. aeruginosa* infections in the host due to the high production of superoxide in the lungs [224].

In addition to responding to paraquat [222,224], *P. aeruginosa* SoxR is also activated by other redox-active compounds, including pyocyanin, toxoflavin, phenazine, methosulphate, plumbagin, menadione, and menadione sodium bisulfite, indicating that this transcriptional regulator is also involved in the response to several oxidants. Furthermore, its activation by pyocyanin implies that it is also activated by endogenous metabolites, showing that SoxR in *P. aeruginosa* is involved in other cellular processes [222]. In this regard, Dietrich and colleagues (2006) showed that the expression of *mexGHI-ompD*, PA2274, and PA3718 was induced by pyocyanin, reinforcing that the activation of SoxR is a protective mechanism against not only paraquat but also to the endogenous production of pyocyanin. The activation of SoxR by pyocyanin occurs under anaerobic conditions, in which there is no formation of superoxide, and in a superoxide-independent manner [226].

#### 3.3.6. Other Transcriptional Regulators

In addition to the transcriptional regulators described in this section, *P. aeruginosa* stress response studies have also identified other regulatory proteins involved in oxidative stress response. Among them, *PA2206* is a novel transcriptional regulator that, along with OxyR, belongs to the LysR-type transcriptional regulators (LTTRs). *PA2206* mutant was significantly less tolerant to exogenous oxidative stress and less lethal in a zebrafish model than the wild-type. Furthermore, the oxidative stress response orchestrated by PA2206 is OxyR-independent and involves the direct regulation of *PA2214-15*, *pvds*, and *PA4881* (gene with unknown function) and approximately 56 other genes, including polyamine metabolism and iron genes [227]. PqrR, a repressor of the *pqrABC* operon [228], and AnvM (anaerobic and virulence modulator) [229] are also involved in oxidative stress response. PqrR and PqrA were also shown to be induced by H_2_O_2_, confirming its role in bacterial survival under peroxide stress [102]. AnvM regulates several genes involved in different pathways, such as oxidoreductase activity, transcription regulation, motility, the response to oxidative stress in the QS system, and pathogenicity [229].

### 3.4. Oxidative Stress Response Contributes to Antibiotic Resistance of P. aeruginosa

*P. aeruginosa* is intrinsically resistant to several classes of antibiotics, mainly due to low outer membrane permeability and the expression of several efflux pumps [15]. Bacterial efflux systems are classified into five families: resistance-nodulation-division (RND) family, major facilitator superfamily (MFS), ATP binding cassette (ABC) superfamily, small multidrug resistance (SMR), and multidrug and toxic compound extrusion (MATE) family. Of these, the RDN family is most commonly associated with antibiotic resistance in *P. aeruginosa* [15,230].

Many of the stress responses elicited by bacteria to counteract the damage caused by oxidative agents contribute to bacterial virulence and resistance, enhancing their survival in the host and the environment. In this context, the oxidative-induced expression of efflux systems has been linked to antibiotic resistance in *P. aeruginosa* [231,232]. MexR, a member of the MarR family that negatively regulates the expression of the efflux system *mexAB-oprM* and *mexR*, is activated upon peroxide oxidation by the formation of a disulfide bond between Cys-30 and Cys-62, which induces a conformational change in the protein and provokes its dissociation from the DNA promoter of the *mexAB-oprM* operon [231]. *mexXY* expression depends on the gene PA5471 (*armZ*) and is involved in the synthesis of the MexXY-OprM system, contributing to aminoglycoside resistance [232]. Nitrosative stress and pentachlorophenol exposure also induced the expression of efflux systems (i.e., *mexEF-oprN* and *mexAB-oprM*, respectively) [233,234].

Moreover, *P. aeruginosa* cells exposed to oxidative stress are often unable to repair all the DNA damage, which increases the mutation frequency in the bacteria, and induces antibiotic resistance mainly due to the increased production of β-lactamase and increased activity of the efflux system MexCD-OprJ [235]. MexAB-OprM, MexXY-OprM, and MexCD-OprJ are important efflux proteins associated with antibiotic resistance in *P. aeruginosa* [15]. They confer resistance to several classes of antibiotics, such as β -lactams, quinolones, tetracycline, and macrolides [15,236,237]. MexXY-OprM is the only system that has been shown to pump aminoglycosides [15,236]. Therefore, oxidative stress acts as a signal for the expression of the efflux system, which contributes to antibiotic resistance in bacteria.

In addition to the stimulation of efflux pumps, oxidative stress can also induce antibiotic resistance by other mechanisms. NaOCl provokes several damages to membrane structures, which favors the transfer of antibiotic-resistant genes from NaOCl-killed bacteria to other injured bacteria in that environment [238]. Furthermore, the induction of biofilm formation is also considered an adaptive response of bacteria against oxidative stress (Reviewed in [32]). Strempel and collaborators (2017) showed that NaOCl induced the expression of cyclic-di-GMP, a second messenger involved in the transition from planktonic to sessile lifestyle, by inducing the diguanylate synthase PA3177, which, therefore, induced biofilm formation [239,240]. This gene was also associated with antimicrobial resistance in *P. aeruginosa* biofilms [241].

Studies have shown that the enzymes that are involved in the oxidative stress response also contribute to antibiotic resistance. In this context, Xia and colleagues (2019) showed that oligoribonuclease (Orn) is required for bacterial resistance to aminoglycoside and β-lactam antibiotics and tolerance to oxidative stress by the regulation of the expression of *katA* at the post-transcriptional level [242]. Orn also contributes to ciprofloxacin tolerance by the overexpression of pyocin [243]. Orn is an exonuclease that hydrolyzes nanoRNAs, which are very short RNAs that contain up to four nucleotides and can be used as primers in the initiation of transcription, resulting in severe alteration in gene expression [244]. It was shown to be required for the expression of the T3SS and bacterial virulence in a mouse acute pneumonia model [245]. Furthermore, Orn also plays essential roles in biofilm formation by inducing the intracellular accumulation of c-di-GMP [246].

AmpR, a major LTTR transcriptional regulator that controls the expression of the β-lactamase *ampC* [247], was shown to positively regulate QS genes, as well as several other genes involved in *P. aeruginosa* antibiotic resistance, extracellular products such as alginate and pyoverdine, and the central metabolism. AmpR also regulated small and antisense RNAs, iron uptake by regulating the siderophores pyoverdine and pyochelin expression, heat shock, and oxidative stress. In this regard, AmpR was shown to positively regulate oxidative stress genes, such as the *katA*, *oxyR*, and *nuo* genes [135].

Finally, the induction of a non-culturable state by the prolonged (36h) exposure of *P. aeruginosa* to H_2_O_2_ provoked a decrease in cell turbidity and cultivability of the bacteria [248]. This non-culturable state is also attributed to antibiotic resistance and a tolerance to harsh environmental conditions [32].

## 4. Final Remarks

The exceptional physiological capabilities of *P. aeruginosa* allow it to survive in harsh conditions and outgrow other bacteria in resource-limited environments [249]. Furthermore, the persistence and prevalence of *P. aeruginosa* in clinical settings are explained by the high metabolic capacity of this pathogen, which gives *P. aeruginosa* the ability to adapt to diverse environments [250]. For example, *P. aeruginosa* can metabolize several carbon sources and use different electron acceptors [251]. These factors, combined with its high resistance to several classes of antibiotics and disinfectants, increase the incidence and severity of *P. aeruginosa* infections, and therefore, the morbidity and mortality rates. It has become a public health concern, and in 2017, the World Health Organization classified this Gram-negative pathogen as group 1 priority for the development of new antimicrobials [252].

In its natural environment, *P. aeruginosa* is continuously exposed to oxidizing agents. Therefore, the survival and successful prevalence of *P. aeruginosa* in these environments are strictly associated with its ability to develop an arsenal of adaptive mechanisms to counteract the damage caused by these agents. Although several studies have been conducted to evaluate the adaptive repertoire developed by this pathogen to counteract oxidative stress, the complexity and robustness of these responses are still in their infancy, mainly because many of these mechanisms are interconnected with other cell processes. For instance, studies have shown that carbon metabolism pathways, post-transcriptional modifications, quorum sensing, and pigment production also play roles in protecting *P. aeruginosa* against oxidative stress.

Among the oxidative stress responses described in *P. aeruginosa*, the expression of detoxifying enzymes, mainly catalases and alkyl hydroperoxides, can be considered one of the most important and prevalent responses under oxidative conditions since their synthesis is controlled by several systems, such as QS and diverse transcriptional regulators. Among the main transcriptional regulators, OxyR is considered the major regulator of oxidative stress responses and has also been shown to regulate genes involved in bacterial virulence. Furthermore, other regulators, including OhrR, OspR, and IscR, have also been shown to possess important roles in defending *P. aeruginosa* against the irreversible damage caused by oxidizing agents. Recently, many researchers have concentrated efforts on studying regulatory pathways and transcriptional regulators involved in *P. aeruginosa*’s oxidative stress responses. However, considering that a large amount of its genome is composed of transcriptional regulators, for example, for PAO1, approximately 10% of its genome is composed of these proteins [99], there are still many adaptive mechanisms to be elucidated.

Understanding the defense mechanisms adopted by this model pathogen would shed light on the development and improvement of new therapeutic options to treat *P. aeruginosa* infections, in which the pathways employed to resist oxidative stress could be targeted by antimicrobials. For instance, azithromycin and macrolides have been shown to downregulate the expression of lipotoxins, such as LptF, and alter biofilms, respectively [253,254]. Furthermore, the glyoxylate shunt, which is not present in humans [255], can also be a potential target for developing new antibiotics. Since these structures are also used as stress responses against oxidative stress, the effectiveness of these antibiotics could be enhanced when treating lung infections of CF patients, in which a high concentration of oxidizing agents is produced, and *P. aeruginosa* is frequently the causative agent. Furthermore, since many of the stress responses are interconnected to virulence factors, such as the upregulation of pigments and conversion to a mucoid phenotype, the elucidation of these mechanisms could improve vaccine and drug development by compromising the virulence potential of *P. aeruginosa* [104].

## Data Availability

Not applicable.
